# Opportunities and challenges for gut microbiome studies in the Indian population

**DOI:** 10.1186/2049-2618-1-24

**Published:** 2013-09-17

**Authors:** Sudarshan Anand Shetty, Nachiket Prakash Marathe, Yogesh S Shouche

**Affiliations:** 1 Microbial Culture Collection, National Center for Cell Science, Ganeshkhind, Pune 411007, Maharashtra, India

**Keywords:** Gut microbiome, Indian population, Diversity, Genetics, Diet

## Abstract

The gut microbiome is a complex ecosystem that affects the development, immunological responses and nutritional status of the host. Efforts are being made to unravel the complex interaction between the gut microbiome and host to have a greater understanding about its role in human health. Colonization of the gut by microbes begins at birth, but the succession and composition of the microbial community depends on a number of factors including, but not limited to, the age, diet, genetic composition, gender, geographic location, and health status of an individual. Therefore, inclusion of diverse human subjects in the study of the gut microbiome is indispensable. However, conducting such studies in India presents unique opportunities and challenges. The vast diversity in human genetic composition, dietary habits, and geographic distribution that exists in the Indian population adds to the complexity in understanding the gut microbiome. Gut microbiome-related studies from other parts of the world have reported a possible association of diseases such as obesity and diabetes with the human gut microbiome. In contrast, an in-depth assessment of risk factors associated with altered gut microbiome in such diseases in the Indian population is lacking. Studies including the Indian population may give insights into the association of the gut microbiome with various factors and diseases that may not be possible from studies on western populations. This review briefly discusses the significance of the gut microbiome on human health and the present status of gut microbiome studies in the Indian population. In addition, this review will highlight the unique opportunities and challenges for gut microbiome studies in the Indian population.

## Review

### Introduction

The human gastrointestinal tract is a unique ecosystem that harbors a diverse population of microorganisms. The association of this microbiome with the host is an important factor that contributes to the overall health of the host.

The interaction of the host and its gut microbiome is a complex one; right from birth various factors shape the composition of the gut microbiome of an individual. At birth, microbes colonize the gut and the mother of the host plays a role as a source of this inoculum [1]. After colonization, the succession and composition of the gut microbiome depends on a variety of factors including the age, diet, genetic composition of the host, gender, geographic location, and health status of an individual [2-7]. These factors result in the development of the indigenous microbiota. Hence, the observations in one group of individuals may not necessarily be applicable to another group, particularly if the individuals have a different genetic make-up, dietary habits and lifestyle. One of the requisites for studying the association of the gut microbiome with various health conditions is inclusion of individuals from diverse populations. Studies on diverse human populations may help in understanding the role of the indigenous gut microbiome in health.

Increasing evidence suggests that dysbiosis exists in the gut microbiome of individuals with various lifestyle-associated diseases such as diabetes [8]. For many years Indians followed a lifestyle that was impervious to the influence of a western/modern lifestyle. There were lesser incidences of diseases like obesity and diabetes that are commonly associated with a western lifestyle. However, the recent changes in socioeconomic conditions have led to a change in the lifestyle of Indians. This change in lifestyle has made the Indian population vulnerable to many metabolic diseases such as coronary heart disease, diabetes, and obesity [9]. In addition to overnourishment-related diseases like obesity and diabetes, India is also facing a severe problem of malnourishment. All these factors are associated with changes in the gut microbiome, thereby affecting the health of the host. A recent metagenomic study on European females with normal, impaired and diabetic glucose control indicated that the metagenomic biomarkers were different from the previously reported biomarkers in Chinese individuals [8]. This study highlighted the need to study individuals from different age groups and geographic locations for identifying appropriate metagenomic biomarkers. However, very few studies on gut microbiota from the Indian population have been conducted, subsequently resulting in a lack of knowledge regarding the association of various diseases and the gut microbiome. Thus, studies on the gut microbiome of the Indian population will help filling the lacunae in knowledge regarding role of gut microbiome in health.

India offers a unique scenario for human microbiome studies. The enormous diversity in genetic composition, dietary habits and ethnicity in the Indian population has interested anthropologists and geneticists for decades. Within the Indian population a high level of population substructure exists, which is represented by four major components: 1) Paleolithic people (indigenous tribals); 2) early farmers from the fertile crescent region; 3) the Indo-European speakers (from central Asia); and 4) the Austro-Asiatic and Tibeto-Burman speakers [10]. Studies on mitochondrial DNA, Y-chromosome, and autosomal loci suggest an influence of west Eurasians in the genetic composition of Indians [11-13]. However, the Indian population differs in the physiology from western populations; the YY-paradox suggests that Indians have approximately three times the fat percent of a western individual with a similar body mass index (BMI) [14].

On account of the heterogeneity of the Indian population, the observations of gut microbiome studies in other populations are not likely to be similar for Indian population. Gut microbiome studies in the Indian population will help in understanding the changes in the gut microbiome under various diseased conditions and the possible impact of these changes on the health of Indians. Furthermore, studies on the gut microbiome in the Indian population are expected to yield new insights with respect to various aspects such as genetics, cultural affiliations, dietary habits and changing lifestyle.

### Gut microbiome and importance in human health

#### Gut microbiome development

Microbes inhabit various body sites in humans and outnumber our own body cells. Of these sites, the human gut is one of the most densely populated anatomical sites. The human gut harbors bacterial, micro-eukaryotic, and viral populations [7]. The most abundant microbial populations are two bacterial phyla, *Firmicutes* and *Bacteroidetes*, and more than 90 percent of the phylotypes identified in gut metagenomic studies are assigned to these phyla [15]. The other bacterial phyla in human gut include *Proteobacteria*, *Actinobacteria, Fusobacteria* and *Verrucomicrobium*.

At birth, the mother is believed to be the source of microbial inoculum, and hence, the mother’s health is considered to have an impact on the microbial composition of the child’s gut [16-18]. The gut microbial diversity gradually undergoes varied compositional changes until it stabilizes at the end of the first year of life after birth [19]. For the rest of the life this dynamic and complex microbial community is constantly under selective pressures (internal pressures such as inter- and intra-organism competition and external pressures like diet, antibiotics and other therapeutics) that have an effect on the evolution of the members of gut microbiome. Ley *et al*. suggested that the structure of microbial community in the human gut is a result of natural selection, which operates at two levels: that is, ‘top-down’ and ‘bottom-up’ selection [20]. The top-down selection is the result of host-driven factors that help distantly related microbial members to evolve convergently for functionally similar genes while bottom-up selection helps microbes specialize for specific functions within the gut [20]. Thus, along with variation in gut microbial populations, the changes in the entire gene repertoire harbored by microbial populations are also observed in association with various factors.

#### Microbial gene repertoire in the human gut

Metagenomic studies have helped unravel the complex gene repertoire that exists within the human gut. The non-redundant microbial gene set is expected to be 150 times larger than the human gene complement [21]. Genes coding for central metabolic pathways, production of amino acids, biosynthesis of vitamins and cofactors, degradation of xenobiotic compounds, etcetera, are reported to form this complex gene repertoire [21]. Thus, the gut microbiome is equipped with a metabolic potential equivalent to a virtual organ within the gastrointestinal tract and hence, considered as an ‘exteriorized organ’ that contributes to the metabolic capability of the host [22,23]. This metabolic capability includes various functions that the human host is incapable of carrying out. For example, the human genome encodes fewer than 20 complex carbohydrate-utilizing enzymes, which indicates that humans cannot digest most of the carbohydrates present in their diet. The microbial populations within the gut serve this important function by degrading the complex plant polysaccharides from sources such as vegetables into simple products that the human body can digest [24]. The gut microbiome serves various functions that have an effect on different aspects of its host health.

#### Influence on human health

Gut bacteria have a profound influence on the development of humoral components of the gut mucosal immune system. They play a crucial role in the development of T-cell repertoires and their cytokine profiles [25,26]. In addition, the microbial ecosystem of the gut serves numerous important functions such as protection against pathogens, nutrient processing, stimulation of angiogenesis, and regulation of host fat storage [27-32]. Short chain fatty acids produced by the gut microbiome help evade colonization of pathogenic bacteria and act as an energy source for colonic epithelial cells [33]. Similarly, microbes produce vitamins, that cannot be synthesized by the host; but these are important for strengthening immunity. It is evident that the gut microbiome influences the host physiology, and dysbiosis leads to impaired functionality of the gut microbiome, resulting in adverse effects on the health of humans.

An alteration of the gut microbiome is reported in physiological conditions such as diabetes. There is a higher proportion of *Bacteroidetes* observed in individuals with diabetes [34]. A recent study reported that patients with type 2 diabetes were characterized by a reduced abundance of some common butyrate-producing bacteria and an increase in various opportunistic pathogens, along with an enrichment of other microbial functions conferring sulphate reduction and oxidative stress resistance [35]. The observation of enriched function related to oxidative stress was noteworthy, as oxidative stress level is considered as one of the factors related to a predisposition for diabetic complications [36]. Thus, the importance of gut microbiome in the pathophysiology of type-2 diabetes cannot be underestimated.

Similarly, obesity and anorexia are also associated with the alteration of the relative composition of some gut microbes [27,37]. Turnbaugh *et al.* identified changes in the gut microbiome as an additional factor contributing to the pathophysiology of obesity [38]. In obese microbiomes an enriched gene set for increased capacity for energy harvest from various sources was observed [38]. On the opposite spectrum of obesity is anorexia. Individuals with anorexia are known to harbor an altered microbiome where *Methanobrevibacter smthii* is dominant in the gut of anorexic individuals [37].

In addition, some autoimmune diseases such as inflammatory bowel disease (IBD) and celiac disease (CeD) are also being studied with respect to their association with the gut microbiome. Researchers have shown that the gut microbiome plays a role in pathogenesis of inflammatory bowel disease by invading the gut mucosa [39]. A comparative metagenomic analysis of fecal DNA of obese individuals and individuals with inflammatory bowel disease has revealed topological shifts in the gut microbiome [40]. Both obesity and IBD are significantly associated with genes for the phosphotransferase system, production of NO_2,_ and metabolism of choline and p-cresol. These are hypothesized to be indicative of the fundamental causes or as a response of gut microbiome to obesity or IBD [40].

For a long time celiac disease (CeD) was thought to be associated with genetic make-up (human leukocyte antigen genes *HLA-DQ2* and *HLA-DQ8*) of the individual and presence of gluten in diet as a trigger for development of CeD in these individuals. Approximately 95% of the patients inherit the alleles encoding for the *HLA-DQ2* and *HLA-DQ8* molecules, but only a small percentage develop CeD [41]. This has led to the hypothesis of a possible role of other environmental factors especially the gut microbiome in pathogenesis of CeD [42]. It is observed that children with celiac disease harbor a more diverse microbiome while there is an increase in abundance of pathogenic bacteria in CeD patients [43]. This suggests a potential role of the microbiome in CeD; however, detailed studies are required to ascertain this association.

#### Studies on gut microbiota in the Indian population

Gut microbiome research in India has been carried out with respect to bacterial succession, mode of delivery, age, obesity, ulcerative colitis, Crohn’s disease, and malnourishment (Table 1). Most of the studies comparing the differences in gut microbial populations in Indians have employed first generation sequencing techniques and/or alternative molecular techniques like qPCR. Pandey *et al*. investigated the effect of mode of delivery (vaginal delivery (VB) and Cesarean section (CB)) on gut microbiome in infants (at day 7 after birth) [44]. It was observed that *Acinetobacter* sp., *Bifidobacterium* sp. and *Staphylococcus* sp. were dominant in the gut of VB infants whereas CB infants had *Citrobacter* sp., *Escherichia coli* and *Clostridium difficile* as the dominant bacteria. Furthermore, the vaginally born infants showed lower species richness than the Cesarean babies. The interesting observation was the high prevalence of *Acinetobacter* sp., which is a clinically relevant bacterial species in VB infants. This needs further detailed investigation.

**Table 1:** Gut microbiome studies in the world population and in the Indian population

**Gut microbiome studies**^**a**^	**World population other than in India**	**Indian population**
**Healthy**		
Infants	Ref: [1,18,45]	Ref: [44,46] (Preliminary studies based on clone library analysis)
Age	Ref: [3,7]	Ref: [47,48] (Specific bacterial groups were targeted); 50 (Included only six individuals from two families)
Diet	Ref: [2,7]	Ref: [49] (Preliminary results based on real-time qPCR)
Geographic	Ref: [2,7,50,51]	No reference available
Genetic	Ref: [4,6]	No reference available
**Disease**		
Obesity	Ref: [27,38,40]	Ref: [52,53] (Results based on clone library and real-time qPCR)
Irritable bowel disease	Ref: [40,54,55]	Ref: [56] (Results based on real-time qPCR)
Diabetes	Ref: [8,34,35]	No reference available
Celiac Disease	Ref: [42,43,57-59]	No reference available

^**a**^Only studies on human subjects have been considered. ref, reference.

Bacterial succession in the colon during different stages of life has been studied in individuals from a south Indian village [47]. *Lactobacillus* and *Bifidobacterium* species were observed to be decreased in adults, whereas there was an increase in *Bacteroides, E. rectale*, and *F. prausnitzii* during late childhood in this population. This was a cross-sectional study where interpersonal differences were not considered. A detailed follow-up study will give a better representation of changes in bacterial diversity with increasing age, that is, from childhood to adolescence. A metagenomic approach in this aspect will give enhanced insights into the variations in gene contents (if any) that occur during transition from childhood to adolescence.

A recent study investigated microbial diversity in the guts of two Indian joint-families with three generations living under the same roof [48]. This study aimed at understanding age-related changes in the gut microbiota. With the effect of diet, genotype, and environmental factors minimized, the authors considered age as the major variable within the same family that may have an effect on gut microbiome composition. This assumption was valid because a recent study by Song *et al*. showed that the individuals co-habiting have a shared microbiome [60]. The pattern of change of *Firmicutes* to *Bacteroidetes* ratio with age observed in this study was different from that observed in other populations around the world. Moreover, 27% of the isolates from the gut of the individuals in this study were potentially novel bacteria. This suggests that the gut microbiota of Indian individuals perhaps is unexplored for novel bacteria, which may include some novel probiotic bacteria. The observations of this study were based on Sanger sequencing of 16S rRNA gene clones and the number of phyla detected were fewer compared to what has been reported with the use of high-throughput sequencing. Previously, it was observed that host-driven factors might lead to distantly related microbial members to evolve convergently for functionally similar genes [61]. As the members of the same family are genetically related and lived together, it would be interesting to explore whether these individuals have a microbiome with shared functionality.

Additionally, infectious diseases and metabolic diseases have also been studied in Indian individuals with respect to compositional variation in the gut microbiome. A study on fecal anerobic commensal bacteria in acute diarrhea samples from children revealed that the members of *Bacteroides-Prevotella-Porphyromonas*, *E. rectale, L. acidophilus*, and *F. prauznitzii* groups were present in low quantities during acute diarrhea compared with levels after host recovery from diarrhea [62]. Similarly, the association of gut microbiota with rotavirus infection, quantitative differences in intestinal *Fecalibacterium prausnitzii* in obese Indian children, and the gut microbiota of Indian women with iron deficiency anemia has also been investigated [46,52,63]. These studies provide important insights into the variation of specific bacterial groups in different health conditions; however, studies that are more detailed are required to better understand the associations of the gut microbiome and the possible role of these associations in such health conditions.

Obesity is a rising concern in India. Patil *et al.* conducted a study on the association of gut microbiota with obesity in the Indian population and reported that *Bacteroidetes* dominated the gut microbiome of surgically treated obese and non-treated obese Indian individuals [53]. This study employed use of clone libraries for the ribosomal small subunit based diversity analysis, eukaryotic genetic markers and real time-based detection of specific bacterial groups. A metagenomic approach for studying the association of the gut microbiome and obesity is required to elucidate variation in gene content in obese, treated obese and lean Indian individuals.

The problem of malnourishment in children is a major challenge in India. Gupta *et al.* demonstrated a possible dysbiosis in the gut microbiome of malnourished children [64]. The malnourished child gut microbiome had an abundance of enteric pathogens belonging to family *Campylobacteraceae* and *Helicobacteraceae*[64], thus, suggesting the susceptibility of malnourished children to colonization by pathogenic bacteria. This was the first study that employed a metagenomic approach to evaluate the gut microbiome of a malnourished Indian child. The gut microbiome of a malnourished child and a healthy child differed both in microbial diversity as well as in the gene repertoires. It was observed that the gut of a malnourished child was characterized by a higher prevalence of virulence-associated genes and a lower prevalence of carbohydrate-associated genes. However, the observations of this study were based on a single subject in each group. Thus, a study with a larger sample size is required to demonstrate a significant correlation between gut microbiome and malnourishment in Indian children. Nonetheless, this study highlights the need for detailed studies that involve children from various socioeconomic backgrounds, geographically distinct locations, and diverse age groups. Such studies may help us to understand if the gut microbiome plays a role in children having reduced capacity for absorption of nutrients.

One disease that has received a great deal of attention in western populations is inflammatory bowel disease. A study on inflammatory bowel disease patients from India revealed that mucosa-associated bacteria such as *Lactobacillus*, *Ruminococcus*, and *Bifidobacterium* were less abundant in these patients. In healthy individuals, the *Bacteroides* group was abundant, whereas a significant decrease was observed in patients with ulcerative colitis and Crohn’s disease [56]. This study targeted certain groups of bacteria and the reason for overall differences in total microbial community remains largely unknown. High-throughput sequencing of 16S rRNA gene or whole shotgun metagenomics can give insights into lesser known microbial taxa and gene repertoires.

In addition to exploring bacterial diversity, researchers have attempted to explore the micro-eukaryotic populations in infants and healthy mothers. While the investigators were able to detect and examine the micro-eukaryotic diversity in the guts of mothers, they did not observe any PCR-detectable micro-eukaryotes in infants. The dominant micro-eukaryotes observed were *Blastocystis* sp. and *Saccharomyces* sp. followed by *Candida albicans*[65]. A metagenomic study of fecal samples from European infants has detected low levels of fungal, viral and archaeal sequences [19]. This makes a persuasive point for exploring the overall gut microbiome using next generation sequencing to provide a holistic view of the Indian infant gut microbiome and how it is evolving as it is exposed to different environmental conditions and varied levels of medical care in rural and urban parts of this developing country. The studies discussed above are preliminary studies that focus on the gut microbiome of Indian individuals. These studies lack sampling efforts, and the use of high throughput technologies is required to verify many of the observations of these studies. Whole shotgun metagenomics and metatranscriptomic approaches with an appropriate sample size may reveal the genetic repertoire in the gut of Indians. Nonetheless, these studies highlight the need for comprehensive studies that assess the role of gut microbiome in various aspects of health and disease in the Indian population.

#### Opportunities for gut microbiome studies in the Indian population

**Figure 1:**
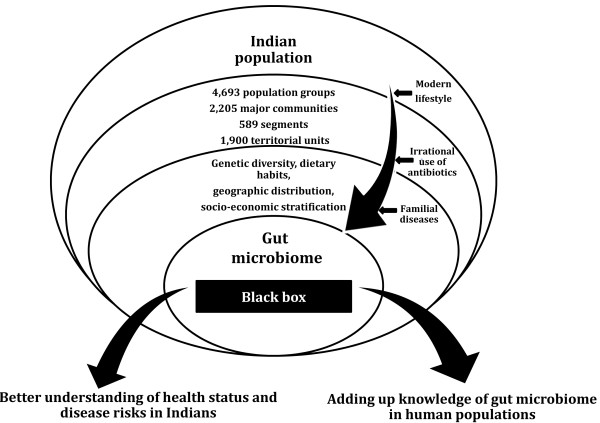
**Potential for human gut microbiome studies in the Indian population.** The Indian population comprises a diverse human population with varied dietary habits, with varied socioeconomic stratification, and with increasing risk from a changing lifestyle to various metabolic diseases. The gut microbiome of this diverse population is still relatively unknown, and a better understanding can be helpful for the healthy life of these individuals.

As discussed in the previous section, knowledge about the gut microbiome in the Indian population is miniscule, thereby presenting numerous opportunities for gut microbiome studies (Figure 1). In addition, the uniqueness of this population provides subject groups to study gut microbiome with respect to various aspects such as genetics, geography, diet and various diseases.

#### Ethnic and genetic diversity

Host genotype is one of the major factors affecting composition of the gut microbiome. A study by Zoetendal *et al.* showed that monozygotic twins had higher similarities in microbial communities than dizygotic twins or unrelated individuals [66]. Hence, it is imperative to study individuals with different genetic make-up to have a better understanding of the association of gut microbiome and host genotype. In this aspect, the Indian population provides one of the most genetically diverse human populations.

The Indian population is broadly classified into four ethnic classes: Australoid, Negrito, Mongoloid, and Caucasoid. The Caucasoid group is spread all over the country, with specific concentration in the northern regions, whereas the Australoid group is mostly restricted to the western and southern states. The Negrito ethnic group is found in the Andaman Islands. Siddis, a migrant group from Africa, resides in Karnataka, Gujarat and Andhra Pradesh. In addition, the Austro-Asiatic tribal groups like Korkus, Mundas, Santhals, Khasis, Nicobarese, Oraon, etcetera, are believed to have migrated to the Indian subcontinent in primeval times [67]. This high genetic diversity in population provides a huge and challenging population base for defining the core gut microbiome. Studies that include such populations may help in elucidating the association between gut microbiome and host genotype.

Migration by humans has led to a large genetic admixture and hence most of the modern population has mixed genetic make-up. In contrast, the Indian population, where the caste system has been prevalent for a long time, has the world’s longest-surviving social hierarchies [50,51]. Marriages within the same caste have promoted the endogamous nature of different groups. For example, one of the genetically homogeneous communities in India is the Parsi community. This community is believed to be more closely related to the European population than most other Indian communities. Whole genome sequencing of the individuals from this population is being done to discover all the polymorphisms in this unique population that potentially bridge the eastern and western populations [68]. Medical research in this population is expected to yield useful information for the development of vaccines [69]. This population is an endogamous population that is declining at a rate of 10% every decade. To preserve the community’s unique culture and heritage the UNESCO has initiated a project (Project 302 IND 70). One of the key questions in gut microbiome research is to understand the reason for the differences in the microbiome of western and the non-western populations. Since the Parsi community is closely related to the European population, especially with the old order Amish population, a comparative study between these two may give insights into the role of factors other than host genetics that shape the gut microbiome in populations in different parts of the world.

The gut microbiome has evolved with different lifestyles, diet, socioeconomic conditions, and the use of various medications by the host. The interesting proposition will be to explore the microbial composition of our ancestors and study the effect of modernization on present-day humans. Recently, an attempt was made to understand the ancient microbiome. In this study, two paleofecal samples originating from cave deposits in Durango, Mexico a representative of the pre-Columbian times dating to approximately 1300 years ago, were examined for their microbial composition [70]. However, a more suitable study group is required to have a better understanding of the evolution of the ancient gut microbiome and the effect of modern lifestyle on the composition and function of gut microbiome. Tribal populations in India provide appropriate subjects for studying the relation of ancient lifestyle and the gut microbiome. Endogamous tribal groups represent genetically isolated populations [71-73]. These isolated tribal populations provide an ancient representation of the human population in terms of the lifestyle and diet. Furthermore, the tribal populations provide a ‘virgin’ avenue for gut microbiome studies as these groups have hardly been exposed to modern medicine and lifestyle. These populations can provide insights into the evolution of the gut microbiome in humans and provide clues regarding the effect of modernization in shaping the gut microbiome.

#### Geographic distribution

Geographic location is a major factor that affects the lifestyle and dietary habits of individuals. This is mainly because the staple foods mostly include the crops and vegetables that are easily cultivated under certain climatic conditions. India is the seventh-largest country (in terms of area) in the world. India is blessed with enormous geographical diversity with the Himalayas on the north, the desert in the west, the northern Ganges fertile plains, the Deccan plateau in the south, and coastal areas and islands [74,75]. People residing in these diverse geographical regions have adapted to specific dietary habits and form a cluster of populations with exposure to specific environmental conditions.

Studies assessing the impact of geographic location on the gut microbiome have shown distinct differences among the gut microbiome of individuals [2,7]. In 2011, a study evaluated infant stool microbiome signatures in two Asian populations, Singapore and Indonesia, with contrasting socioeconomic development and examined the putative influences of demographic factors on these human fecal-associated bacterial signatures [76]. This study demonstrated the associations of geographical origin with *Clostridium leptum*, *Atopobium* and *Bifidobacterium* groups. A comparative study of the gut microbiome of Korean individuals showed a distinct gut microbiome composition when compared to individuals from the United States, Japan and China [77]. Such studies are still lacking in India.

In addition to microbiome composition, the difference in the gene content harbored by the microbiome has been studied in different populations by Yatsunenko *et al.*[7]. One of the major genes that varied according to geographic location of the individuals was the urease gene [7]. The Malawian and Amerindian baby microbiome had a significantly higher representation of this gene compared to the U.S population [7]. Thus, it would be interesting to ascertain which bacteria and genes are present in Indian infants from geographically different locations.

Knowledge of the gut microbiome in relation to geographic location in the Indian sub-continent, and particularly in the Indian population, is lacking. The enormous diversity in geographic conditions present throughout the length and breadth of India provides opportunities for studying various aspects of the gut microbiome with respect to geographic location.

#### Diversity in dietary habits

The co-evolution of the gut microbiome with the host is driven by host diet [60]. The diversity of the intestinal microbiome increases from carnivory to omnivory to herbivory. Present-day humans living a modern lifestyle have microbial communities typical of omnivorous primates [78]. Therefore, it is necessary to understand how different dietary habits of humans from different parts of the world have shaped the gut microbiome.

In India, various ethnic groups have diverse dietary habits. This diversity in diet is not comparable to the diversity of diets in western populations. The diversity in diet of the Indian population gives a unique identity to this culturally diverse group. The diet compositions of Indians vary according to their socioeconomic status as well as by their caste and religious affiliation [45,79]. A large population of Hindus, especially the Brahmin and Jain families, follow a strict vegetarian diet that does not include egg, meat and fish. Kabeerdoss *et al*. compared the gut microbiome of South Indian women who follow either vegetarian or omnivorous diets. They observed that the *Clostridium* cluster XIVa bacteria, specifically *Roseburia-E. rectale,* were present abundantly in the fecal microbiome of the omnivorous group [49]. The strict vegetarian Indian population provides study groups not found in many parts of the world. Since ancient times, several generations of the individuals from these groups have been on diet devoid of meat. These populations can serve as ideal subjects for studying the effect of diet in evolution of the gut microbiome compared to the other humans that consume meat. It will be interesting to explore whether the diversity of microbial populations is higher in vegetarians compared to non-vegetarians.

Recently, a group studied the effect of diet and health in elderly individuals of Caucasian (Irish) ethnicity [80]. They observed that the diet-based groupings are associated with separations in the microbiome. This study suggested that there is an underlying relationship between diet, microbiome and health, which essentially means that studies on the gut microbiome related to diet in various disease conditions may be able to show a correlation among these three factors. Individuals on vegetarian diets and non-vegetarian diets may have the same disease. The insights into such diet-microbiome-health relationships may be obtained by studying Indian individuals with differing dietary habits. Moreover, metagenomic studies can help elucidate the gene repertoire of gut microbiome of these individuals. It is logical to expect that the gut microbiome of vegetarian individuals may have a higher gene content related to degradation of complex plant polysaccharides. This, however, will be clear only with a well-designed experiment that employs metagenomics and metatranscriptomics as tools for unraveling the genetic potential of the microbiome.

The Indian diet involves a vast variety of spices and some spices used in daily diet have proven antibacterial activity [81]. For example, clove, cinnamon and mustard are spices used in everyday diets, and these spices have exhibited antibacterial activity against clinical isolates [82]. The effect (if any) of these spices in shaping the gut microbiome is largely unexplored. Many aspects of the microbiome related to diet have not been studied in the Indian population. Thus, there is an opportunity to explore the effect of these dietary habits on the gut microbiome. These studies may help in better understanding the role of diet in shaping microbiome composition.

#### Disease risks of Indian population

There is an increasing prevalence of chronic diseases globally, and, although it is mostly associated with developed countries, the developing countries are not far behind. Many of the chronic diseases in developing countries are associated with changing lifestyle. The prevalence of diseases such as irritable bowel disease and celiac disease, along with various life-style associated diseases such as obesity, diabetes and cardiovascular disease has increased in the Indian population in the last few years [83-88]. Indians exhibit distinctive features such as excess body fat, abdominal adiposity and increased fat at various body sites and thus, are considered being at a risk to developing obesity-related disorders [14]. In spite of this, it is intriguing that there is varied prevalence of obesity in urban and rural Indians. The urban population has a higher prevalence of obesity compared to the rural population [89]. If these individuals are equally at a risk of developing these disorders, what is the reason for the observed difference in prevalence? Are there other environmental triggers that lead to the development of this condition? In this aspect, determining a possible role of dysbiosis on the microbiome as a result of different lifestyles is required for gaining further insights into development of obesity in Indians.

Obesity is associated with and considered a major factor leading to metabolic syndrome and type-2 diabetes mellitus. Hence, considering the epidemic of obesity, it is not surprising to find an increasing epidemic of diabetes in India [90]. Although, diabetes is considered a modern disease associated with modern lifestyle, it is an ancient disease in the Indian population. Reference to this disease as *madhumeha* exists in some ancient texts [91]. While the genetic basis of type 2 diabetes mellitus in Indians is the same as in Caucasians (that is, a strong association of TCF7L2 variants with type 2 diabetes), there are other factors that make this population a unique cohort [92]. It is hypothesized that Indians have the so-called ‘Asian Indian Phenotype’, which involves a genetic predisposition to diabetes and premature coronary artery disease [93]. While a basal metabolic index (BMI) of 23 kg/m^2^ is considered a healthy BMI in European population, Indians with this BMI have an increased risk of hyperglycemia [94]. This suggests that Indians are prone to diabetes at a lower BMI compared to the European population. Indians with same BMI as Europeans have higher body fat percentage and hence, Indians are described as ‘thin-fat Indian’ [14]. Considering these differences between Indians and Europeans, it is logical to expect that studies related to these disorders in Europeans or for that matter, any other population worldwide, contribute little understanding to the role of the gut microbiome in the health status of Indians. It becomes imperative to study the gut microbiome of Indian individuals to ascertain the role played by the gut microbiome in these diseases.

Some of the diseases, for example, celiac disease and diabetes, have familial links. Family members exposed to same diet and environmental factors are difficult to study in western populations as these populations include nuclear families. The concept of the joint family is prevalent in India, where many generations of a family stay together under the same roof [95]. The influence of environmental factors on variability of the gut microbiome will be minimal in such cases. Moreover, the dietary habits are more or less similar. Thus, these individuals in joint families can be considered as ideal subjects for studying the role of gut microbiome in diseases that have familial links.

In addition to the genomic approaches, classical microbiological studies are also required in gut microbiome studies of the Indian population. These studies may help in isolation of indigenous bacteria that can be developed as probiotics. The most studied bacterial genera for its probiotic features is *Lactobacillus*. These bacteria are known to have varied specificity of adhesion for human epithelial intestinal Caco-2 and Int-407 cell lines [96]. Thus, bacteria isolated from a western population may not necessarily be successful in colonizing the gut of Indians. Moreover, the gut microbiome is a complex ecosystem, and different selective pressures exist depending on host-associated factors such as genetics and other factors such as dietary habits and lifestyle. Thus, the bacteria in the ecosystem evolve differently, presenting stiff competition for survival of foreign bacteria. Efforts have been directed toward isolation and development of indigenous probiotic strains for Indians [97,98]. One of the *Lactobacillus* strains, that is, *Lactobacillus plantarum* Lp9, is under study for its probiotic features. This bacterium demonstrated high resistance to low pH, bile, antibacterial, antioxidative, and cholesterol lowering properties [99]. However, a more comprehensive clinical validation of these strains in the target Indian population is necessary. As there is no consensus on the bacteria that are commonly present in Indian individuals, the research has been targeted towards specific bacterial groups. The microbiome analysis of Indians may help elucidate the bacterial populations that are commonly encountered in the gut of Indians. This may lead towards the development of novel indigenous probiotics.

#### Irrational use of antibiotics

Apart from the above mentioned physiological diseases, India also has one of the highest numbers of bacterial diseases [100]. The use of antibiotics is a lifesaver in severe bacterial infections. However, irrational use of antibiotics is common in India. Many patients undergo antibiotic treatment for common cold and other conditions where antibiotics are avoidable. It is now a well-known fact that bacteria develop resistance to antibiotics, subsequently causing severe complications in treatment of bacterial infections. Moreover, evidence suggests that with increased use of antibiotics, the human gut microbiome may act as a reservoir of antibiotic resistance when antibiotics pass through the colon [101]. External factors such as antibiotics have an impact on the composition of the gut microbiome. The most commonly used antibiotics are of a broad range that kill pathogens as well as the commensal and ‘beneficial bacteria’ and thus have an impact on the overall microbial community dynamics in the gut. One commonly used antibiotic, ciprofloxacin, influences nearly one-third of the bacterial population and results in reduced diversity and evenness of the community [102]. In elderly individuals, antibiotic treatment has an adverse effect on the gut microbiome making individuals vulnerable to pathogenic infection [103].

Irrational use of antibiotics is rarely observed in western populations. Thus, we hypothesize that the core ‘Indian gut microbiome’ might have evolved to be resistant to various antibiotics compared to the ‘western gut microbiome.’ The misuse of antibiotics may have selected microbes that have evolved and adapted to a high antibiotic usage. For evidence, a comparative study of the gut microbiome in healthy western individuals and Indian individuals has to be carried out.

Undoubtedly, India is home to a diverse population with matchless features and distinct identities that separates them from the rest of the world. This population provides avenues that are very difficult (if not impossible) to explore in other populations. Additionally, the distinct identity of Indians calls for gut microbiome studies related to various aspects of health, and these studies need to be conceptualized with ‘Indian’ as the focal point. Although such studies will be challenging they will bring understanding that is relevant to the health of Indians.

#### Challenges for gut microbiome studies in Indian population

Although, this population presents enormous potential for gut microbiome research, several challenges remain to be overcome. Firstly, defining a sample size that represents more than a billion Indians seems a daunting task in itself. The massive diversity means that the definition of a core microbiome will be a difficult proposition. This will require a study unmatched in terms of sample size and sequencing effort. A thorough understanding of the Indian population will require collaborative studies that involve geneticists, anthropologists, and biologists. The sequencing and computational technologies would be a bottleneck in large-scale correlation analysis between the diverse microbiome harbored by Indians.

Secondly, legislative laws in India protect tribal populations that would provide a source of ‘virgin’ microbiome that is untouched by modern medicine. The other major challenge will be communicating with these tribal groups, as language will be a major barrier and convincing these individuals an even more difficult task for a researcher. Our tribal brothers will definitely give insights into our ancient microbiome, but these studies will require a sensitive and humane approach from researchers, as these individuals are a special fraction of our society.

Thirdly, correlating the gut microbiome with diet will require extensive data on dietary habits. The composition of each of the food components needs to be assessed. This will require maintaining thorough metadata regarding dietary habits.

The correlation of dietary habits and lifestyle with diseases such as obesity and diabetes is well known. However, the different populations that develop these diseases may vary in daily dietary intake. Therefore, associating diet-gut microbiome-disease will require large sampling that includes various fractions of this vast population. Furthermore, longitudinal studies that involve detailed follow-up of individuals that are at a risk of developing a certain disease is essential. Such studies will require a meticulous effort by researchers who will also be assessing the gut microbiome in order to detect any biomarkers associated with the microbiome in such individuals.

As Indians are at risk of developing obesity and diabetes, a study with highly controlled evaluation of genotype, lifestyle (rural populations with ‘Asian Indian Phenotype’ and urban individuals with the same phenotype but with modern lifestyle) has to be carried out. This is possible with Indian populations as there is a fair difference between the rural and urban population in terms of lifestyle and dietary habits. Such studies are necessary to have a comprehensive view of the microbiome and its association with disease state. Nevertheless, such challenging studies will help elucidate the relation of different dietary habits to gut microbiome.

Finally, there is a need to create awareness regarding the gut microbiome and its impact on health among the Indian population. This awareness will lead to active participation from larger fractions of the population leading to comprehensive studies. This may underpin the role of the gut microbiome in various diseases.

## Conclusions

The Indian population, which encompasses a vast diversity of individuals, is yet to be studied extensively for the gut microbiome. The second largest population of the world provides challenging, interesting, and resourceful populace for studying the gut microbiome. Various ethnic groups and tribal populations hold potential for population-based studies concentrating on genetics and geographic influences on the gut microbiome. Because the gut microbiome is affected by several intrinsic factors such as genetics of the individuals, the study of gut microbiome on a genetically diverse population will give deeper insights on effect of such factors on the gut microbiome.

The other important factor that affects the gut microbiome is the lifestyle of individuals. India is undergoing a rapid transition from a developing country to a developed country. In addition, globalization has assisted in the increased movement of Indians to other parts of the world. It is expected that this level of globalization may result in loss of some key features of the evolutionary histories of our microbiome by the virtue of homogenization of human-associated microbial communities [104]. Thus, extensive sampling of our human microbiome, particularly in societies that are undergoing dramatic cultural, socioeconomic, and technological transformations, is crucial. The study of the gut microbiome in the Indian population may help in elucidating this transformation of the gut microbiome and identify the key features that may be lost after transition to the modern lifestyle.

Even today, India harbors some tribal populations that follow the ancient lifestyle. This means that the key features of the microbiome prior to a complete change to a modernized society may also be preserved. A comparative study between these populations and other world populations may also help in having a closer representation of gut microbiome prior to modernization in these populations. The rapid changes in socioeconomic conditions pose a great challenge that requires a huge effort to conduct these studies as soon as possible, before we lose this key information.

Because the gut microbiome plays an important role in the health of humans, the knowledge regarding its association with various diseases is necessary. It is speculated that different populations may have different biomarkers associated with a specific disease [9]. In an Indian context the increased prevalence of diseases like cardiovascular disease, celiac disease, irritable bowel syndrome, obesity, and diabetes necessitates immediate attention of researchers around that world and especially from Indian researchers to understand the gut microbiome of this population.

Gut microbiome-related studies are also important for the development of indigenous probiotic strains. These strains have to be tested for their probiotic potential in different diseases and cater to the needs of Indians. Thus, a comparative study of the gut microbiome, including healthy and diseased subjects, will ultimately be crucial to gain a better understanding about the health of Indians.

Overall, India provides a unique study group for gut microbiome studies with respect to various factors. However, the Indian population also presents its own distinctive challenges that have to be addressed when gut microbiome studies are being conducted. Nevertheless, the knowledge obtained from studies including the Indian population may lead researchers toward an enhanced understanding of the significance of the gut microbiome in human health.

## Abbreviations

CB: Cesarian delivery; CeD: Celiac disease; IBD: Inflammatory bowel disease; VB: Vaginal delivery.

## Competing interests

The authors declare that they have no competing interests.

## Authors’ contributions

SS and NM contributed equally toward the completion of manuscript. YS guided and assisted in writing the manuscript. All authors read and approved the final manuscript.
